# Nonrandom Selection and Multiple Blood Feeding of Human Hosts by *Anopheles* Vectors: Implications for Malaria Transmission in Papua New Guinea

**DOI:** 10.4269/ajtmh.21-0210

**Published:** 2021-09-27

**Authors:** John B. Keven, Michelle Katusele, Rebecca Vinit, Daniela Rodríguez-Rodríguez, Manuel W. Hetzel, Leanne J. Robinson, Moses Laman, Stephan Karl, David R. Foran, Edward D. Walker

**Affiliations:** ^1^ Department of Microbiology and Molecular Genetics, and Department of Entomology, Michigan State University, East Lansing, Michigan;; ^2^ Vector-borne Diseases Unit, Papua New Guinea Institute of Medical Research, Madang, Papua New Guinea;; ^3^ Department of Epidemiology and Public Health, Swiss Tropical and Public Health Institute, Basel, Switzerland;; ^4^ Department of Epidemiology and Public Health, University of Basel, Basel, Switzerland;; ^5^ Vector-Borne Diseases and Tropical Public Health Group, Burnet Institute, Melbourne, Victoria, Australia;; ^6^ Division of Population Health and Immunity, Walter and Eliza Hall Institute of Medical Research, Parkville, Victoria, Australia;; ^7^ Department of Medical Biology, University of Melbourne, Melbourne, Victoria, Australia;; ^8^ Australian Institute of Tropical Health and Medicine, James Cook University, Cairns, Queensland, Australia;; ^9^ School of Criminal Justice and Department of Integrative Biology, Michigan State University, Michigan

## Abstract

Nonrandom selection and multiple blood feeding of human hosts by *Anopheles* mosquitoes may exacerbate malaria transmission. Both patterns of blood feeding and their relationship to malaria epidemiology were investigated in *Anopheles* vectors in Papua New Guinea (PNG). Blood samples from humans and mosquito blood meals were collected in villages and human genetic profiles (“fingerprints”) were analyzed by genotyping 23 microsatellites and a sex-specific marker. Frequency of blood meals acquired from different humans, identified by unique genetic profiles, was fitted to Poisson and negative binomial distributions to test for nonrandom patterns of host selection. Blood meals with more than one genetic profiles were classified as mosquitoes that fed on multiple humans. The age of a person bitten by a mosquito was determined by matching the blood-meal genetic profile to the villagers’ genetic profiles. Malaria infection in humans was determined by PCR test of blood samples. The results show nonrandom distribution of blood feeding among humans, with biased selection toward males and individuals aged 15–30 years. Prevalence of *Plasmodium falciparum* infection was higher in this age group, suggesting males in this age range could be super-spreaders of malaria parasites. The proportion of mosquitoes that fed on multiple humans ranged from 6% to 13% among villages. The patterns of host utilization observed here can amplify transmission and contribute to the persistence of malaria in PNG despite efforts to suppress it with insecticidal bed nets. Excessive feeding on males aged 15–30 years underscores the importance of targeted interventions focusing on this demographic group.

## INTRODUCTION

In Papua New Guinea (PNG) where malaria is endemic,^1^ long-lasting impregnated bed nets (LLINs) that target the mosquito vectors are the primary malaria intervention method, supplemented with an increased supply of rapid immunologic diagnostic test kits and antimalarial drugs at local health centers.^2^^–^^4^ Immediately after nationwide implementation of the LLIN-based malaria control program in 2005, a considerable decline in the rates of malaria transmission and infection was observed nationally.^5^^–^^8^ However, in recent years (2016–2017) infection rates have either persisted (plateaued) or rebounded despite high LLIN coverage.^9^^,^^10^ Physiological or genetic resistance to the pyrethroid insecticides in the LLIN has not been detected in the *Anopheles* vector populations in PNG.^11^^–^^13^ However, decreased bioefficacy of the LLINs distributed between 2013 and 2019 against pyrethroid-susceptible vectors in natural populations in PNG has been observed and is believed to be a factor causing persistent malaria transmission in PNG.^14^ Decreased bioefficacy is a result of poor quality of the LLINs and not of physiological or genetic resistance to them. The phenomenon of persistent transmission in the presence of high LLIN coverage in PNG might also be caused by mosquito behaviors such as outdoor and early evening human-biting and opportunistic host selection, which enable mosquitoes to evade the indoor-deployed LLINs.^15^^–^^17^ In addition, multiple and nonrandom feeding on human hosts by the vectors can increase the transmission potential of malaria.^18^^–^^24^ Multiple feeding refers to mosquitoes that obtain a blood meal from more than one source, usually thought of as different vertebrate species, for example, human, pig and dog hosts for PNG mosquitoes.^25^ However, multiple feeding can occur within a host species too, involving, for example, multiple human sources.^26^ Nonrandom human feeding refers to the situation where some individuals in a human community are fed on by mosquitoes more frequently than others.

Investigation of multiple and nonrandom human feeding involves analysis of mosquito blood meals. Molecular methods have been widely used to identify vertebrate host species fed on by mosquitoes and have enabled the evaluation of important aspects of mosquito–host interaction, especially host selection and resulting vector–human contact patterns.^25^^,^^27^^–^^36^ This strategy also allows for a better understanding of the effectiveness of vector control measures.^25^ In recent years, analysis of mosquito blood meals has been extended to include multilocus genotyping of microsatellite markers that serve as unique genetic profiles (“DNA fingerprint”) that identify or differentiate individuals of a host species, particularly humans in blood-meal samples.^26^^,^^37^^–^^43^ This has opened up the possibility of investigating multiple and nonrandom human feeding by *Anopheles* vectors of malaria.

Genetic profiling of mosquito blood meals has three important applications in studies of malaria transmission. Firstly, if the mosquito biting rate is nonrandom so that some individuals in a community are bitten more frequently than others, these individuals are more likely to be infected and serve as reservoirs or even sinks of infections.^18^^–^^22^^,^^24^ Thus, identification of such high-risk individuals or population groups and their characteristics can inform strategies, particularly those applied in resource-limited countries, to design targeted disease interventions. For example, in villages without an ongoing malaria control program, distributing interventions such as antimalarial prophylactic drugs or LLIN primarily to those segments of the human population who are bitten the most could reduce prevalence of infection in parasite reservoirs and thereby diminish rates of transmission, simultaneously increasing cost-effectiveness of the intervention.^44^^–^^48^ In villages where there is an ongoing control program, population groups that are not protected from mosquito bites can be identified.

Secondly, assessing patterns of mosquito-biting frequencies among individuals is important to prognose the course of disease epidemiology, particularly in the context of a disease control program. Malaria prognosis is usually based on two epidemiological quantities: (i) basic reproduction number (*R*_0_), which is the number of new human infection cases in a susceptible population that arises as a result of transmission from a single primary case throughout its infective life and (ii) the vectorial capacity (*V*), which is the expected number of infective bites that arises from all the *Anopheles* that bite a single infectious person in a single day.^49^ Studies have shown that the values of both *R*_0_ and *V* under nonrandom conditions of vector–human contact, whether socially or spatially, are higher than under random conditions.^19^^,^^20^^,^^22^^,^^24^^,^^50^ As malaria transmission increases with *R*_0_ and *V*, such nonrandom blood feeding patterns may permit transmission to persist with potential for resurgence, even when a vector control program is underway.^22^

Thirdly, the proportion of blood-fed mosquitoes that obtain multiple blood meals can be estimated. Estimating the proportion of multiple blood meals is epidemiologically important for the following reason. A female mosquito can lay several batches of eggs at different times during her lifetime, with each egg-laying event preceded by a blood meal.^51^^,^^52^ Each blood feeding–egg laying event is called a gonotrophic cycle. During a gonotrophic cycle, a mosquito that takes a blood meal from a single human can infect only one person if the mosquito is sporozoite-positive or, if it is malaria-free, has only one chance of becoming infected depending on the person’s gametocyte infection status. By contrast, a mosquito that takes a blood meal from multiple humans can infect more than one person and has multiple chances of contracting an infection in a single gonotrophic cycle, thereby increasing both *R*_0_ and *V*.^23^ Host defensive behavior is one important cause of multiple blood meals as it interrupts mosquitoes during blood feeding, causing them to complete a full meal on a different human host.^53^

In this study, genetic profiles of human hosts, based on genotyping of 23 microsatellite loci and a sex-specific marker, were analyzed from blood samples of village residents and blood meals of female *Anopheles* vectors of malaria in PNG. The data were used to quantify the proportion of blood-fed mosquitoes that fed on multiple humans and assess nonrandom pattern of human host selection by the vectors. The general methodological approach of genetic profiling is summarized schematically in Supplemental Figure 1 (Supplemental file 1). The risk of malaria infection in over-selected and under-selected humans was also evaluated.

## MATERIALS AND METHODS

### Study sites.

**Figure 1.**
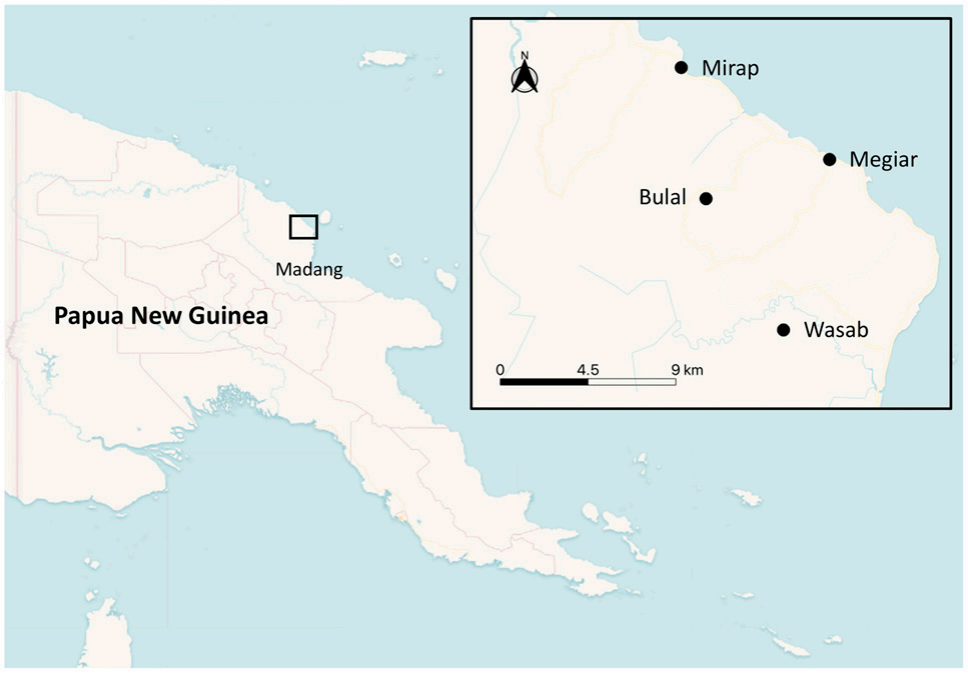
Map of Papua New Guinea showing location of the study villages in Madang province. This figure appears in color at www.ajtmh.org.

This study was conducted in Bulal, Megiar, Mirap and Wasab villages in the north coast region of Madang province, PNG (Figure 1) where malaria is endemic.^1^^,^^8^^,^^54^ Megiar and Mirap are situated on the coastal plain adjacent to the ocean, elevated about 1.5–2.5 m above sea level. Bulal and Wasab are situated several kilometers inland from the coastline on elevated hilltops about 150 m above sea level. The topographic and vegetation features of the coastal and inland environments where these villages are located are described elsewhere.^55^

### Mosquito sampling and identification of blood-meal hosts.

Mosquitoes were collected for 6 nights in Bulal (March 16–21, 2017), Megiar (February 4–9, 2017), and Mirap (January 11–16, 2017), and 12 nights in Wasab (September 5–10 and November 4–9, 2016) using the barrier screen method.^56^ Mosquito sampling involved five or eight barrier screens per village per night and local volunteers (villagers) were consented and trained as mosquito collectors. The mosquito collectors were given antimalarial chemoprophylaxis to protect them from infectious mosquito bites. The structure and setup of the barrier screen equipment and the procedure for collecting mosquitoes on the barrier screens are described elsewhere.^55^ Blood-fed *Anopheles* were separated from unfed and non-*Anopheles* species with light microscopy and stored dry on silica gel desiccant in the field. The mosquitoes were then brought to the laboratory where the abdomen of each mosquito was separated from the rest of the body and extracted for genomic DNA using DNeasy Blood and Tissue Kits (catalog number, 69582; Qiagen, Valencia, CA). *Anopheles* species were identified by a method involving PCR amplification of the internal transcribed spacer region 2 of the ribosomal RNA gene followed by endonuclease digestion of the amplicons, which produces species-specific DNA fragment patterns on agarose gels.^57^ The blood-meal origin was identified by a multiplex real-time PCR assay with host-specific probes, including human, that target specific nuclear or mitochondrial DNA loci.^58^ Mosquitoes whose blood meals originated from animal hosts, excluding those with a mix blood meal from animal and human hosts, were excluded from further analysis.

### Human blood sampling and malaria detection.

In the same week that the mosquitoes were collected in the villages, capillary human blood samples (250 μL) were obtained from a subset of consented village residents representing different age groups (age range, 0.5–85 years old) and from all the mosquito collectors working in the villages. Consent for minors was obtained from parents or guardians. A multiplex real-time PCR containing two fluorophore-labeled TaqMan probes targeting the 18S ribosomal RNA gene of *Plasmodium falciparum* (forward primer: ATT GCT TTT GAG AGG TTT TGT TAC TTT; reverse primer: GCT GTA GTA TTC AAA CAC AAT GAA CTC AA; probe: FAM-CAT AAC AGA CGG GTA GTC AT-MGB) and *Plasmodium vivax* (forward primer: GCA ACG CTT CTA GCT TAA TCC AC; reverse primer: CAA GCC GAA GCA AAG AAA GTC C; probe: VIC-ACT TTG TGC GCAT TTT GCT A-MGB)^59^ was optimized for sensitivity in tests with positive control samples. The tests detected as low as one target gene copy/μL sample and the amplification efficiency was greater than 90%. The optimized reaction mixture (10 μL volume) consisted of 1x TaqMan Multiplex Master Mix (catalog number, 4481882; Thermo Fisher Scientific, Waltham, MA), 0.6 μM of each primer, 0.4 μM of each probe, and 2 μL of DNA samples. PCR reactions were performed on a QuantStudio 7 Flex instrument (Applied Biosystems, Foster City, CA) with fast cycling consisting of one cycle of 95°C for 20 seconds followed by 40 cycles of 95°C for 1 second and 60°C for 20 seconds. Genomic DNA was extracted from 200 μL of each human blood sample using the NucleoMag^®^ Blood 200 μL kit (catalog number, 744501.1; Macherey-Nagel, France) and subjected to the real-time PCR described previously to test for malaria infection. Human-fed mosquito blood meals were also subjected to the real-time PCR to test for malaria infection in the engorged blood. Samples with amplification threshold cycle ≥ 38 were inconclusive and considered negative. Although human blood and mosquito samples were obtained from all four villages, only the samples of Mirap and Wasab were analyzed in the malaria detection assay. As hypothesis testing involving human genetic profiles necessitates evaluation of patterns of malaria infection, lack of human genetic profiles in Bulal and Megiar (see the next paragraph for reason) rendered malaria detection unnecessary in these two villages.

### Human genotyping.

Human multilocus genotypes (genetic profiles) of the village residents including the mosquito collectors who donated blood samples during the malaria infection survey and the *Anopheles* blood meals were analyzed using a microsatellite genotyping assay used in forensic identification.^60^ While the mosquito blood meals of all four villages were analyzed, human blood samples of Mirap and Wasab only were analyzed. Human blood samples of the other two villages were not analyzed because blood samples of the mosquito collectors, which were essential for hypotheses testing involving genetic profiles (see Results and Discussion), were not obtained. The assay used a multiplex PCR kit (PowerPlex Fusion System; catalog number, DC2402; Promega, Madison, WI) that contained fluorophore-labeled primer pairs that co-amplified 22 autosomal microsatellite loci (D3S1358, D1S1656, D2S441, D10S1248, D13S317, Penta E, D16S539, D18S51, D2S1338, CSF1PO, Penta D, TH01, vWA, D21S11, D7S820, D5S818, TPOX, D8S1179, D12S391, D19S433, FGA, D22S1045), one Y chromosome-linked microsatellite locus (DYS391), and one locus serving as a sex-specific marker (Amel).^60^ The primers that target these human loci do not amplify other vertebrate species commonly used by *Anopheles* mosquitoes as blood hosts except three nonhuman primates (chimpanzee, gorilla, and macaque), which are not found in PNG, although their genetic profiles are easily distinguished from those of humans.^60^ The PCR reaction mixture (10 μL reaction volume) consisted of 6 μL of water, 1 μL of 5x Fusion Master Mix, 1 μL of 5x Fusion Primer Mix, 0.5 μL of 10x PCR Buffer (catalog number, N8080010; Thermo Fisher Scientific), 0.5 μL of 25 mM MgCl_2_ (catalog number, N8080010; Thermo Fisher Scientific), and 1 μL of DNA (for samples with low DNA concentration, 4 μL were used with the water volume adjusted to 3 μL). Thermal cycling consisted of one cycle of 96°C for 1 minute followed by 30 cycles of 94°C for 10 seconds, 59°C for 1 minute, 72°C for 30 seconds, and one cycle of 60°C for 10 minutes. For detection of amplicon size, solution mixtures consisting of 1 μL of the PCR products or Allelic Ladder (catalog number, DG381B; Promega) combined with 0.3 μL of WEN Internal Lane Standard 500 (catalog number, DG5001; Promega) and 9 μL of formamide (catalog number, 4311320; Thermo Fisher Scientific) were analyzed by capillary electrophoresis (3500 Genetic Analyzer; Applied Biosystems). Results were analyzed using GeneMapper software version 4.1 (Applied Biosystems). Microsatellite alleles were represented by the number of sequence repeats, which can be heterozygous or homozygous. The sex-specific marker indicated a female individual if it was homozygous (X, X) or a male if heterozygous (X, Y). For each sample, the genotype of each locus was listed in a Microsoft Excel spreadsheet. In the spreadsheets (see Supplemental files 2 and 3), the alleles of each marker were separated by a comma; for heterozygous genotypes, the alleles were listed in order from small to large. Blood-meal profiles with three or more alleles at multiple loci were considered to be mosquitoes that fed on multiple human hosts (Supplemental file 2).

### Data analyses.

To identify unique genetic profiles, a program written in R (version 3.4.2; https://www.R-project.org/) engaged loop functions and conditional statements to compare the genotype of each locus in a query sequence (i.e., a genetic profile) to its corresponding locus in a subject sequence (i.e., another genetic profile). The similarity of two (query and subject) genetic profiles was expressed as the proportion of identical loci (i.e., same genotype) times 100. For example, 18 identical loci divided by 24 total loci multiplied by 100 gives 75% profile similarity. In principle, genetic profiles from two blood-meal specimens that originated from a same human source will have 100% profile match. Thus, match values < 100% indicate blood meals from different individuals. However, as some blood-meal specimens yield insufficient human DNA (e.g., due to very small mosquito blood meal volume), false mismatches due to “allele dropout”—a failure of the alleles in a locus to be PCR-detected^61^—can arise. To account for this potential error, a value less than 100% was used as the criterion for establishing a match. To determine this value, pairwise percent profile match analysis was performed on the genetic profiles of village residents. This generated *n*(*n*–1)/2 profile match results or values, where *n* is the number of village residents. A value higher than the highest value in the pairwise match output, excluding match results of monozygotic twins, which were 100%, was the criterion below which two genetic profiles were considered different (see Results).

To eliminate collector-feeding bias in the analysis of nonrandom human hosts selection by mosquitoes, blood meals whose genetic profile matched that of any mosquito collector from the village where the mosquitoes were collected were removed from the data. This analysis was performed for blood-meal genetic profiles of Mirap and Wasab only as collectors’ genetic profiles were not available for the other two villages. Blood meals with multiple human sources were not useful for this analysis and were also removed from the data. After excluding the collector-fed blood meals, the number of different human individuals in a blood-meal sample was determined from the number of unique genetic profiles in the sample. The frequency of occurrence of each unique genetic profile in the blood-meal sample represented the number of mosquitoes that fed on each human individual. These data were used to construct frequency distribution histograms that relate the number of different human individuals (*y*-axis) to the number of blood meals they received (*x*-axis). The observed frequency distributions were fitted to zero-truncated Poisson and zero-truncated negative binomial frequency distribution models using the functions *zerotrunc* and *rootogram* of the package *countreg*^62^ in R software. The rational for fitting the observed frequency distribution data to the two statistical models is that randomly distributed discrete data are generated by Poisson processes whereas data that have a clustered distribution, which is a nonrandom distribution are generated by negative binomial processes.^63^ Thus, χ^2^ supported fit of the data to a Poisson distribution indicates random selection of human individuals by the mosquitoes, presumably reflecting no bias, whereas fit to a negative binomial distribution (which also means lack of fit to the Poisson model) indicates nonrandom selection, presumably reflecting bias. Additionally, the observed proportion of blood meals acquitted from human individuals of a specific sex or age group was compared with their expected proportion by the two-tailed exact binomial test. The expected proportion of a specific human group is the proportion of individuals of that group in the random sample of village residents who provided a blood sample along with information about their sex and age. The proportion of a human group calculated from the random sample of residents of a village is an estimate of the true proportion of that group in the human population. As females went to sleep earlier in the night and were more likely to be protected by the bed net than men who remain active outdoors late into the night in the study villages,^64^ the proportion of female-fed relative to male-fed blood meals was expected to decrease in the latter segments of the night. To test this prediction, the proportion of female-fed blood meals in the evening (6 pm–10 pm), night (10 pm–2 am) and morning (2 am–6 am) periods was plotted for Mirap and Wasab blood-meal samples (collector-fed blood meals were excluded) and χ^2^ analysis was used to test for homogeneity of proportions across the three periods.

For Mirap and Wasab villages where malaria detection in human blood samples was performed, the likelihood that different age groups and sexes were infected with *P. falciparum* and *P. vivax* was tested by logistic regression of binary data. As many of the individuals identified in the mosquito blood meals did not provide a blood sample, their infection status was determined based on PCR test of blood meals. These individuals were then categorized into two groups: those whose genetic profile was encountered in a single mosquito blood meal, and those whose profile was encountered in two or more blood meals. Fisher’s exact test was used to determine variation in the prevalence of infection between the two groups. The exact binomial, logistic regression and Fisher’s exact statistical tests were performed using the functions *binom.test*, *chisq.test*, *glm*, and *fisher.test*, respectively, of the R package *stats*. Significance level of all statistical tests was based on type I error rate of 0.05.

### Research ethics.

The procedures for recruiting the study participants and mosquito collectors were reviewed for compliance with human protection and other ethical concerns and approved by Papua New Guinea Institute of Medical Research Institutional Review Board (IRB No. 1517), Papua New Guinea Medical Research Advisory Committee (MRAC No. 16.08), World Health Organization Ethics Review Committee (ERC No. ERC.0002671), and Michigan State University Institutional Review Board (IRB No. 17-1603). Written informed consent was obtained from all participants and mosquito collectors. To compensate for loss of economically valuable time spent working for us, the mosquito collectors were given a small amount of money in local currency at a rate of five US dollars per night. This was approved by the three institutional review boards mentioned previously.

## RESULTS

### Multiple blood meals.

**Figure 2.**
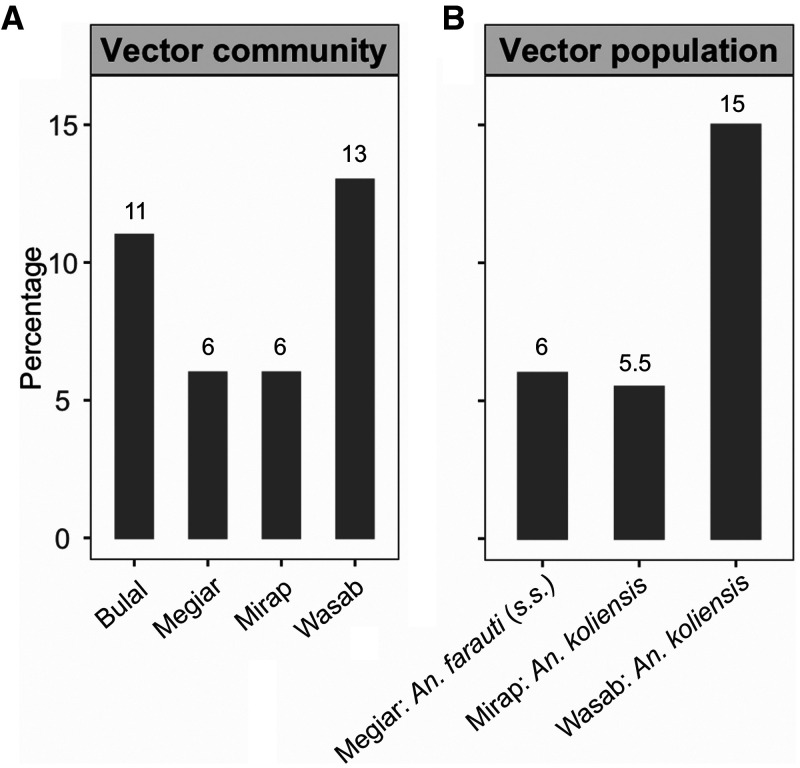
Percentage of mosquitoes with multiple blood meals. (**A**) For *Anopheles* communities in Bulal (*N* = 18), Megiar (*N* = 124), Mirap (*N* = 233), and Wasab (*N* = 378). (**B**) For three *Anopheles* populations: *An. farauti* (s.s.) in Megiar (*N* = 116), *An. koliensis* in Mirap (*N* = 200), and *An. koliensis* in Wasab (*N* = 339). Numbers above the vertical bars are the observed percentages of mosquitoes with multiple blood meals.

A total of 881 *Anopheles* blood meals from the four villages confirmed by host-specific PCR to have originated from human hosts were analyzed for human genetic profiles. Of these blood-meal samples, 85% (*N* = 753) yielded a genetic profile; the other 15% (*N* = 128) either did not amplify in the PCR reaction or had grossly incomplete profiles. Of those samples that yielded a genetic profile, 18 were from Bulal, 124 from Megiar, 233 from Mirap, and 378 from Wasab (Table 1 and Supplemental file 2). It is worth noting that the imbalance in the number of blood-meal samples among the villages was a direct result of natural variation in mosquito abundance in those villages^10^ and not from biased selection of the samples. The distribution of these blood-meal samples according to mosquito species—*Anopheles bancroftii*, *Anopheles farauti* (sensu stricto, s.s.), *Anopheles koliensis*, *Anopheles longirostris*, and *Anopheles punctulatus* (s.s.)—is presented in Table 1. The proportion of blood-fed mosquitoes with blood meals from multiple human sources is shown for *Anopheles* spp. (i.e., regardless of species) in each village (range: 6–13%) in Figure 2A and for three vector populations with sample size > 100 (range: 5.5–15%) in Figure 2B.

**Table 1:** Distribution of successfully genotyped blood meals according to mosquito species

*Anopheles* species	Bulal	Megiar	Mirap	Wasab
*An. bancroftii*	0	0	1	0
*An. farauti* (s.s.)	0	116	31	3
*An. koliensis*	15	8	200	339
*An. longirostris*	0	0	1	0
*An. punctulatus* (s.s.)	3	0	0	36

### Nonrandom human host selection.

The tests of nonrandom selection of human hosts were performed on mosquitoes from Mirap and Wasab only. These tests were not applied to mosquitoes from Bulal and Megiar as it was not possible to eliminate collector-fed blood meals from these two villages because of lack of the collectors’ genetic profiles to match with the blood-meal profiles. Inclusion of collector-fed blood meals in the analysis can bias the results because they are likely to be overrepresented in the samples due to constant presence of the collectors near the mosquito sampling site. Also, the tests were performed on *Anopheles* mosquitoes in general; analysis according to species entailed working with low sample size.

As the tests of nonrandom human host selection involved evaluation of percent match between pairs of blood-meal genetic profiles, a criterion (a percent match value) for deciding a match (or a mismatch) was first established as follows. DNA extracted from blood samples of 440 Mirap residents and 285 Wasab residents were genotyped. Genetic profiles of 95% (*N* = 419; 201 males and 218 females) of the Mirap residents and 58% (*N* = 164; 90 males and 74 females) of the Wasab residents were successfully obtained (see Supplemental file 3). Comparison of each human genetic profile with all other genetic profiles resulted in 87,571 and 13,366 pairwise percent profile match values for Mirap and Wasab, respectively. For both villages, these results consisted of 18 unique percent match values, each with a varying number of occurrences. The distribution of the probability (proportion) of occurrence of the unique values is shown in Supplemental Figure 2 (Supplemental file 1). For both villages, the lowest percent match value was 0% with 0.04 probability of occurrence. This means that 4% of all the pairwise comparisons were between individuals with no identical genotype at any locus. The highest percent match value was 71% (i.e., 17/24 matched loci) in both villages with probability of occurrence of 10^−5^ and 10^−4^ for Mirap and Wasab, respectively (Supplemental Figure 2, Supplemental file 1). This means that no two humans in both villages had a profile match that was > 71%. Because it is unlikely for any two individuals to be 71% identical, this value could be chosen as the criterion for deciding whether the genetic profiles of two mosquito blood meals originated from the same person. However, this value is not conservative enough as it allows for up to seven false mismatched loci, which is too many. Instead, 79%, which had zero probability of occurrence and allows for at most five false mismatched loci, was chosen. Thus, blood-meal genetic profile pairs that had match values below 79% were considered to originate from different human sources, whereas those with values 79% or greater were from the same human source.

**Figure 3.**
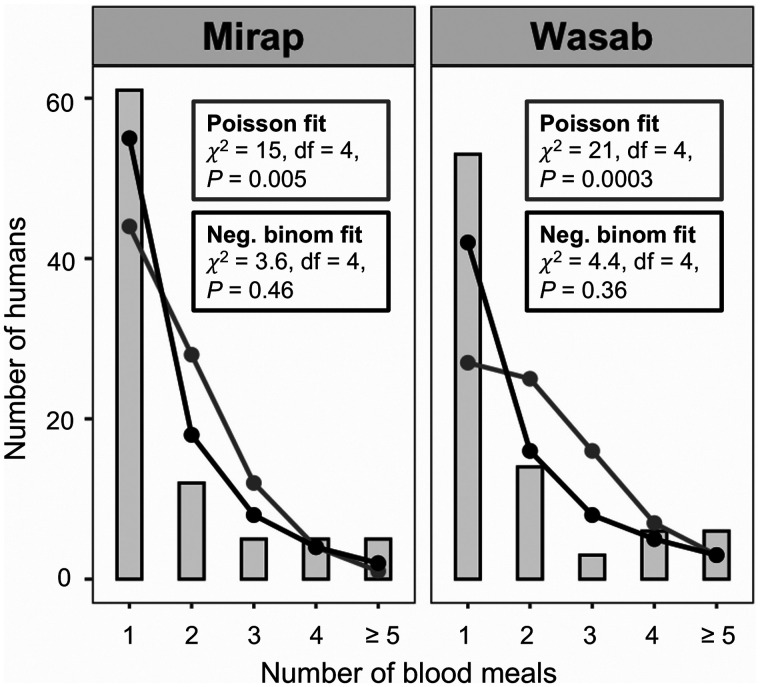
Blood meal frequency distribution. Fits of zero-truncated Poisson (grey curve) and zero-truncated negative binomial (black curve) expected frequency distribution to observed frequency distribution (grey vertical bars) of number of blood meals taken on a human by *Anopheles* spp. in Mirap and Wasab villages.

Of 233 successfully genotyped Mirap blood meals, 94% (*N* = 218) were single-human meals. Of 378 successfully genotyped Wasab blood meals, 87% (*N* = 328) were single-human meals. Of the 218 Mirap single-human blood-meal profiles, 29% (*N* = 63) were collector-fed blood meals. Of the 328 Wasab single-human blood-meal profiles, 46% (*N* = 152) were collector-fed blood meals. After excluding the collector-fed blood-meal profiles from the data and performing pairwise percent match analysis on the noncollector profiles, 88 unique profiles were identified in the Mirap blood meals (*N* = 155) and 82 unique profiles were identified in the Wasab blood meals (*N* = 176). That is, the blood meals of Mirap mosquitoes originated from 88 different human individuals and of Wasab mosquitoes originated from 82 different individuals. For each of the two villages, a histogram was constructed that relates the number of blood meals taken on a person (*x*-axis) to the number of human individuals with a particular blood-meal frequency (*y*-axis) (Supplemental Figure 3, Supplemental file 1). Of the 88 different human individuals identified in Mirap blood-meal sample, most (69%) were fed on by a single mosquito, 25% of people were fed on by two to four mosquitoes, and 6% were fed on by more than four mosquitoes. There were two individuals that were fed on by 10 mosquitoes each (Supplemental Figure 3, Supplemental file 1). Of the 82 different individuals identified in Wasab blood meals, most (65%) were fed on by a single mosquito, 28% of people were fed on by two to four mosquitoes, and 7% were fed on by more than four mosquitoes. There were two individuals who were bitten by 15 or 17 mosquitoes (Supplemental Figure 3, Supplemental file 1). Fits of the two observed frequency distribution to zero-truncated negative binomial and zero-truncated Poisson distributions are shown for both Mirap and Wasab blood meals in Figure 3. χ^2^ results show that the data fit well to the negative binomial, with nonsignificant differences between observed and expected frequency distributions, and poorly to the Poisson model with significant differences between the observed and expected frequency distributions (Figure 3). Fit of the data to the negative binomial distributions indicates nonrandom or clustered pattern of human host selection by the *Anopheles* mosquitoes in both villages.

**Figure 4.**
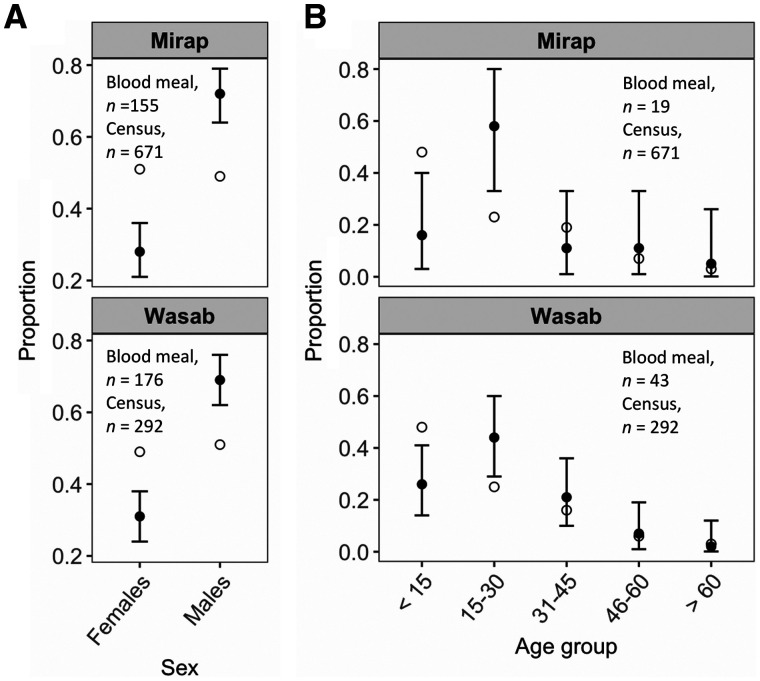
Results of exact binomial tests comparing the observed and expected blood-meal proportion. (**A**) Of each sex group in Mirap and Wasab villages. (**B**) Of each age group in the two villages. The observed blood-meal proportions are represented by black dot with 95% confidence interval bars and the expected proportions are represented by the open circle. The sample size (*n*) of blood meals and human census count for each village are shown in the plot.

The results of exact binomial tests comparing the proportion of *Anopheles* spp. that fed on a female and male person to the proportion of female and male residents of the village are shown for Mirap and Wasab in Figure 4A (collector-fed blood meals were excluded). Despite a nearly equal proportion of the sexes in both Mirap (51% females, 49% males) and Wasab (49% females, 51% males), there were significantly more male-fed (Wasab, 69%; Mirap, 72%) and less female-fed (Wasab, 31%; Mirap, 28%) mosquitoes than expected (Figure 4A). In Mirap, the proportion of female-fed blood meals relative to male-fed ones was higher early in the night (evening) than in the two later periods of the night. In Wasab, the result was opposite; the proportion of female-fed blood meals relative to male-fed ones was lower in the evening than in the latter periods of the night. However, the observed variation in the proportion of female-fed relative to male-fed blood meals among the three periods of the night was not significant (general χ^2^ tests) in Mirap or Wasab (Supplemental Figure 4, Supplemental file 1). Unlike sex, it was not possible to determine a person’s age based on a genetic marker. Thus, the age of a person who was bitten by a mosquito was determined by searching the genetic profiles of villagers (their ages were known) for a match (based on the 79% match criterion) to the blood-meal profile. Twelve percent (19/155) of Mirap and 24% (43/176) of Wasab blood-meal profiles were successfully matched to a village resident; the remaining blood-meal profiles did not have a match because the genetic profile of most of the villagers was not obtained. In both villages, the proportion of blood meals in the three older age groups did not deviate from the expected proportion (exact binomial test), whereas significant deviation was observed in the two younger age groups; blood meals obtained from individuals under 15 years old were underrepresented, whereas those obtained from 15–30 years old were overrepresented in both villages (Figure 4B).

### Risk of malaria infection.

**Figure 5.**
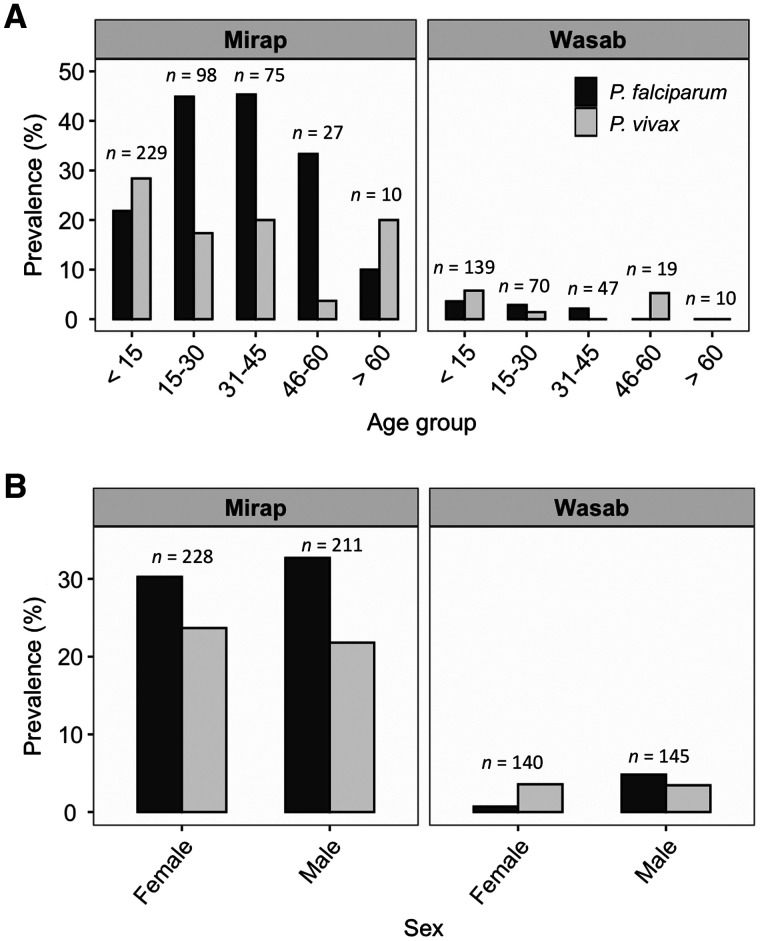
Prevalence of malaria infection in Mirap and Wasab. (**A**) Age-specific *P. falciparum* and *P. vivax* prevalence. (**B**) Sex-specific *P. falciparum* and *P. vivax* prevalence. The sample size (*n*) of each age or sex group is shown above the bar corresponding to the group.

Prevalence of infection with *P. falciparum* and *P. vivax* by sex and age groups in Mirap and Wasab is shown in Figure 5. The results of logistic regression tests comparing the likelihood of infection with *P. falciparum* or *P. vivax* in male individuals relative to females (reference group), and for individuals in each of four age groups relative to those under 15 years (reference group) are shown for Mirap (*N* = 440 study participants) in Table 2 and for Wasab (*N* = 285 participants) in Table 3. Mirap blood samples used in this study (*N* = 440) were from a subset of total study participants who participated in a larger related study focused on malaria epidemiology. Wasab blood samples (*N* = 285) on the other hand were not a subset but the total number of participants reported in the larger study. In Mirap, the likelihood of infection with *P. falciparum* was statistically higher in the age groups 15–30 years and 31–45 years relative to the < 15 years age group; no difference was observed for the two older age groups (Table 2). For *P. vivax* in Mirap (Table 2) and both malaria species in Wasab (Table 3), the risk of infection was the same as or statistically lower but not higher than the < 15 years group. No difference was observed between the two sexes for both malaria species in both villages (Tables 2 and 3). Infection data from mosquito blood meals showed that the prevalence of infection with any malaria species for those human individuals whose genetic profile was detected in a single blood meal was 12.9% (*N* = 62) in Mirap and 3.7% (*N* = 54) in Wasab and for those detected in two or more blood meals was 15.4% (*N* = 26) in Mirap and 8.0% (*N* = 25) in Wasab. The prevalence of infection between the two human groups was the same in both Mirap (Fisher exact test: odds ratio [OR] = 0.83; 95% confidence interval [CI]: 0.2, 4.2; *P* = 0.75) and Wasab (OR = 0.47; CI: 0.03, 6.79; *P* = 0.59).

**Table 2:** Logistic regression results for test of variation in likelihood of malaria infection for individual humans in four age groups relative to those < 15 years (reference group), and for males relative to females (reference group) in Mirap

Reference variable	Variable	β	Std. error	*P*
*P. falciparum*	15–30 years	1.07	0.26	< 0.0001*
	31–45 years	1.10	0.28	0.0001*
	46–60 years	0.60	0.44	0.171
	> 60 years	−0.92	1.07	0.387
	Male	0.15	0.21	0.481
*P. vivax*	15–30 years	−0.64	0.30	0.036*
	31–45 years	−0.47	0.32	0.149
	46–60 years	−2.35	1.03	0.022*
	> 60 years	−0.46	0.80	0.567
	Male	−0.15	0.23	0.510

* Indicate significant *P* value.

**Table 3:** Logistic regression results for test of variation in likelihood of malaria infection for individual humans in four age groups relative to those < 15 years (reference group), and for males relative to females (reference group) in Wasab

Reference variable	Variable	β	Std. error	*P* value
*P. falciparum*	15–30 years	−0.13	0.86	0.883
	31–45 years	−0.39	1.12	0.725
	46–60 years	−16.01	2.3 × 10^3^	0.995
	> 60 years	−16.43	3.3 × 10^3^	0.996
	Male	1.94	1.08	0.072
*P. vivax*	15–30 years	−1.45	1.07	0.177
	31–45 years	−16.78	1.5 × 10^3^	0.991
	46–60 years	−0.11	1.09	0.921
	> 60 years	−16.75	3.4 × 10^3^	0.996
	Male	−0.11	0.65	0.862

## DISCUSSION

Several studies have applied genetic profiling of mosquito blood meals to investigate patterns of contact between human hosts and mosquito vectors of diseases, including malaria vectors.^26^^,^^37^^–^^39^^,^^41^^–^^43^ The current study was the first to apply this method of investigation to *Anopheles* vectors of malaria in PNG. This study also differed from the previous ones in the number of microsatellite loci used to construct a genetic profile. The previous studies had 10 or fewer loci, whereas the present one had 24 loci, meaning the present study had greater power to discriminate between genetically related individuals such as siblings.^40^

An important finding in this study was that the frequency of *Anopheles* blood meals acquired from different humans in the villages was not randomly distributed but rather was clustered. That is, some human individuals were fed on more frequently compared with other members of the communities. The nonrandom pattern of human host selection is not unique to this study as other studies that also used blood-meal genetic profiling approach have observed this phenomenon in both *Anopheles*, *Culex*, and *Aedes* populations and their associated mosquito-borne disease systems.^26^^,^^37^^,^^39^^,^^41^ This study also observed that certain demographic groups were disproportionately over-represented (or under-represented) in mosquito blood-meal samples. In both Mirap and Wasab villages, the *Anopheles* obtained more blood meals from males and individuals of the 15–30 years age group compared with females or other age groups. A striking observation was under-representation of the youngest age group (< 15 years), which constituted a large proportion of the village residents (52% of Mirap, 50% of Wasab), in the blood meals. Similar findings have been reported for populations of *Anopheles funestus* and *Anopheles gambiae* in villages of Tanzania. In that setting, males and individuals ≥ 20 years received more *Anopheles* bites than expected, whereas females and those < 20 years old were bitten less frequently in a village with bed nets, while in a village without bed nets, such variations were not observed.^43^ In a Kenyan village, individuals < 20 years old received more bites than expected compared with those between 20 and 50 years old, but no variation was observed between sexes.^42^ In five Zambian villages, the proportion of male-fed and female-fed blood meals were homogeneous with the expected proportions; data on age groups were not reported.^65^ Epidemiologically, mathematical models of malaria transmission show that nonrandom distribution of vector bites among human hosts increases the *R*_0_, causing transmission to persist^19^^–^^22^^,^^24^^,^^66^ even in the presence of an intervention program such as distribution of LLINs.^22^ Therefore, targeted intervention focusing on those human groups biasedly over-selected by vectors may help greatly in reducing malaria transmission and infection rates.

One likely explanation for the nonrandom vector–human contact pattern is that the number of *Anopheles* in different spatial locations in villages is not randomly distributed but rather aggregated, a condition that was observed in the villages investigated in this study.^10^ Therefore, humans are most likely to encounter female *Anopheles* where the mosquitoes themselves aggregate. Human behavior and LLIN usage could also explain these patterns. For instance, lower bed net use by adult males has previously been found as a general pattern in PNG.^4^ Humans also undertake particular activities during *Anopheles* biting times, often related to their age and gender; not all village residents would, therefore, be equally likely to gather in the location in which mosquitoes aggregate.^64^ A mixed-methods investigation of human behavioral patterns in the same villages investigated here found that pre-school aged children (< 16 years old) go to bed comparably earlier and are most likely to regularly use mosquito nets.^64^ On the other hand, males above 16 years of age were more likely to be exposed to mosquitoes as they go to sleep later in the night and use mosquito nets less often.^64^ This implies that the proportion of blood meals taken on female humans relative to males decreases in the latter periods of the night than in the evening (6 pm–10 pm) as a result of protection from mosquito bites provided by sleeping under bed nets early in the night. However, analysis of this prediction was not statistically supported, perhaps due to masking effects of unknown confounding variables that were not accounted for in the analysis. Generally, sleeping behavior and bed net use in the older people and pre-school aged children is consistent with the risk of bites observed in the two age groups in the present study.

In the malaria transmission system, the human individuals who contribute most of the blood meals could be recognized as “super-spreaders”, as they are likely to be infected and serve as reservoirs of the parasite to be spread to other members of the community.^18^^,^^21^^,^^66^ In villages near those investigated in the current study, people over the age of 20 who were infected with either *P. vivax* or *P. falciparum* malaria commonly harbored gametocytes (*P. vivax*, 32%; *P. falciparum*, 48%).^67^ Thus, it can be inferred that individuals 15–30 years old who are likely to be fed upon at a greater than random encounter rate can be viewed as potential sources of malaria infection to mosquitoes (i.e., super-spreaders) and are thus potential candidates for targeted intervention. However, the term super-spreader is associated with gametocyte density more than frequency of mosquito bites as the likelihood of mosquitoes to become infected after taking an infectious blood meal increases with gametocytemia of the person from whom the blood was imbibed.^18^ Although individuals that are 15–30 years old in PNG are gametocytemic, their gametocyte densities (*P. vivax* geometric mean, 1.9 gametocytes/μL; *P. falciparum* geometric mean, 3.2 gametocytes/μL) are up to 5.5 (*P. vivax*) or 6.7 (*P. falciparum*) times lower than those of younger age group.^67^ The low gametocytemia may render them less infectious to mosquitoes. Apart from super-spreaders, another way of looking at the epidemiological significance of high rates of mosquito bites taken on individuals that are 15–30 years old is to recognize them as “parasite sinks”, which means that these individuals attract most of the mosquito bites including infective ones, but the infections in these individuals do not result in high gametocytemia for them to serve as super-spreaders, thereby minimizing the intensity of malaria transmission.

With regard to risk of infection, the current study found that the prevalence of infection with any malaria species in those individuals who were bitten multiple times was greater than those bitten only once in both Mirap (1.2-fold greater) and Wasab (2-fold greater), which was consistent with the prediction. However, these variations in infection were not statistically significant. Also, based on the observed heterogeneous distribution of vector bites on humans, it was predicted that the likelihood of malaria infection would be higher in male than female individuals and in individuals in the youth age (15–30 years) than the other age groups. This prediction held true for the age group variable in Mirap’s *P. falciparum*; it was not the case for Mirap’s *P. vivax*, nor either parasite species in Wasab, although the prevalence of infection for both malaria species in Wasab was very low. Interestingly, the prevalence of malaria infection for both species did not differ between sexes, which would not follow from observed biases in *Anopheles* feeding.

The consistency between biased feeding and risk of infection with *P. falciparum* among age groups in Mirap village supports the prediction that people who encounter frequent vector bites are more likely to be infected than are those that are bitten less frequently. For *P. vivax* in Mirap and both malaria species in Wasab, a plausible explanation for the inconsistency between biased feeding and risk of infection is that the human biting rates (number of mosquito bites per person per unit time) in the underutilized human groups (i.e., females and nonyouth age groups) were high enough to support transmission across these demographic groups such that the probability of infection was homogeneous in all ages and sexes despite biased mosquito feeding patterns. This assertion is supported indirectly by studies showing that the rate of malaria transmission, which is measured in terms of entomological inoculation rate—the number of malaria infective *Anopheles* bites received per person per year—must be reduced below 1 before appreciable reduction in prevalence of infection in the human population can be observed.^68^^,^^69^ Thus, for *P. vivax* in Mirap and both malaria species in Wasab, the human biting rates must be reduced sufficiently for the variation in infection among human groups with varying frequency of vector bites to be revealed. It can also be revealed by analysis of “multiplicity of infection” through parasite genotyping; individuals with high biting rates are likely to harbor multiple parasite clones from exposure to multiple infectious *Anopheles* bites.^70^ Nevertheless, the blood-meal genotyping method described in this study is sufficient to identify the risk groups in high malaria transmission settings without the need for analysis of multiplicity of infection.

The proportion of mosquitoes with blood meals from multiple human sources observed in *Anopheles* spp. in the four villages ranged from 6% to 13% and in the three *Anopheles* populations with sufficient sample size from 5.5% to 15%. Those results were in the same range as the ones produced in a similar study conducted in another Madang village in which the proportion of mosquitoes that fed on mother–child pairs was quantified using ABO blood group markers.^53^ The study showed that 13% of *Anopheles* spp., 11% of *An. punctulatus* (s.s.), 13% of *An. koliensis*, and 18% of *An. farauti* (s.s.) had multiple blood meals; that is, the mosquitoes fed on both mother and child.^53^ The proportion of multiple blood meals in PNG *Anopheles* observed in both the current and previous studies were similar to those reported for *An. funestus* (2–14%) and *An. gambiae* (0–11%) in two Kenyan villages,^42^ and *An. gambiae* (10%) in a Tanzanian village.^43^ Similar percentages were also observed in *Culex* and *Aedes* populations,^26^^,^^37^^,^^41^ except for an *Aedes aegypti* population in Thailand, where a much higher proportion (45%) was observed.^38^

The sensitivity of hosts to mosquito bites affects the outcome of a blood meal; the more intolerant a host is to mosquito bites, the more likely a blood-feeding attempt will be interrupted before a full blood meal is obtained.^71^^,^^72^ An interrupted mosquito can either forgo further blood-feeding attempts and settle for a partial meal or it can complete the blood meal by feeding on a second individual of the same host species or a different species. Whether a mosquito obtains a subsequent meal on an individual of the same or different host species, both outcomes have important epidemiological consequences. With respect to the former condition, in high malaria transmission settings such as the selected study sites in PNG, *Anopheles* mosquitoes that feed on two human hosts per gonotrophic cycle increase both *R*_0_ and *V* by 2-fold or greater.^23^ This is possible because those vectors can infect more than one human if they are sporozoite-positive. Interruption of blood feeding does not affect the delivery of sporozoites into the human hosts; inoculation of a single sporozoite can result in an infection. One may also argue that feeding on multiple individuals might increase the chance of a mosquito to obtain an infectious (i.e., gametocytemic) blood meal in a single gonotrophic cycle. However, an interrupted blood meal consists of a smaller amount of blood, which means that the probability of imbibing gametocytes from a person is lower than a full blood meal taken on the same person. Thus, both measures of transmission were likely increased by 6–13% in both Mirap and Wasab village as these were the proportions of human-fed vectors that fed on two or more people. With respect to the latter, *Anopheles* mosquitoes that obtain a second blood meal on human hosts after they were interrupted by an animal host increases the odds of infecting humans as well as the odds of being infected by humans, both of which amplify transmission rates. Further, before genetic profiling methods were available, the proportion of mosquitoes with multiple human blood meals was estimated indirectly from mathematical models.^53^^,^^72^ With the availability of genetic profiling method, this quantity can be estimated directly without the need for modeling as demonstrated in the current study.

An important consideration that was taken into account in this study was minimization of errors that can cause false mismatches between pairs of genetic profiles. The primary sources of error are the degree of relatedness of villagers and the frequency of allele dropout per locus. Regarding the degree of relatedness, if villagers are closely related to each other genetically, then the ability to discriminate among blood meals taken from different villagers is reduced, resulting in false matches between blood meal profiles that originated from different human sources. Regarding the frequency of allele dropout, if alleles drop out of the analysis owing to insufficient DNA, then false homozygosity would result in false mismatches of blood-meal profiles that originated from the same human source. One study showed that villagers of New Ireland, New Britain, and Bougainville provinces in PNG, similar to most villages elsewhere in the country including those in the present study, have low genetic diversity and are genetically related to many other members of the village through nuclear and extended family connections.^73^ This means that for most people, the percent profile match among members of the village will be high and thus require a greater number of microsatellite markers to successfully differentiate them. In this study, the pairwise percent profile match based on 24 loci found that the highest percent match between any two nonmonozygotic individuals was 71%, but the probability of observing this value was 10^−4^ or 1 in 10,000 pairwise trials, which is extremely low. This means that a minimum of 17 markers (16 microsatellites and one sex-determining marker), which is similar to the number of markers used in other forensic identification protocols,^74^^–^^76^ is sufficient to discriminate between two individuals from a typical PNG village. Choosing 79% (which had zero probability of occurrence) instead of 71% as the criterion for deciding a match provides a decision rule allowing up to five erroneous mismatched loci of the 24 loci examined here while maintaining the power to correctly discriminate between different individuals. Previous studies involving genetic profiling and matching of blood meals to human individuals did not consider this important element of the identification process and accordingly have not established this quite necessary rule of thumb, nor incorporated these potential sources of error into their analysis.^26^^,^^37^^,^^38^^,^^41^^–^^43^

This study had several limitations. Firstly, it is worth noting the low success of obtaining a genetic profile from analysis of human blood samples of Wasab (58% success rate) compared with Mirap, which was high (95% success rate). This discrepancy was attributed to the quantity of DNA in Wasab samples which was on average 10-fold lower than Mirap samples. The cause of variation in the DNA quantity among the villages is not known but attributed to variation in laboratory conditions; Wasab samples were extracted in a different laboratory than those of Mirap using the same extraction method. This problem can also cause false negative results in malaria detection assay, which may explain the curiously low prevalence of malaria infection in Wasab compared with Mirap village (see Figure 5). Secondly, the genetic profiles were not obtained for all the residents of the study villages as many residents did not consent to donating a blood sample. For this reason, the blood-meal sources of some mosquitoes could not be identified and characterized. However, there is no reason to believe that this problem may affect or bias the analyses and inferences regarding the distribution of mosquito bites among different age groups based on the mosquito samples whose blood meal sources were identified. Thirdly, because of sample size issue, the analysis of multiple blood meals and nonrandom feeding patterns were performed on *Anopheles* spp. in general rather than for each species separately. This can be a problem if different species within a village vary in host-seeking behaviors such as peak biting time and indoor versus outdoor biting pattern. Similar studies in the future should attempt to generate sufficient sample size to evaluate each species separately. Fourthly, the probability of interrupted blood feeding by mosquitoes might vary between the collectors and the general population in a village due to variation in the behavior of these two groups to biting mosquitoes. The collectors would be more aware of the presence of biting mosquitoes and are not engaged in the usual village activities that might promote or detract from the probability of interrupted feeding by mosquitoes. This has the potential to bias the estimates of percentages of mixed blood meals reported previously, which were calculated without eliminating from the data mixed blood meals that contained the collectors’ blood. Given the number of possible human contributors in the village who were likely to share the same alleles with the collectors, it was difficult to determine with certainty whether a collector contributed to a mixed blood meal based solely on the ambiguous, mixed genetic profiles. This analysis is better done by way of high-throughput sequencing of hypervariable region of human mitochondrial D-loop.^33^

In summary, this study demonstrated the usefulness of applying genetic profiling analysis to human-fed mosquito blood meals. By identifying different human sources of mosquito blood meals based on unique genetic profiles, three epidemiologically important features of malaria vectors were able to be investigated. Firstly, the proportion of mosquitoes that amplifies malaria transmission potential by feeding on multiple human individuals within a single gonotrophic cycle was estimated and ranged from 6–13% among villages. Secondly, the level to which blood meals taken on different human individual was randomly distributed was examined and was found to be nonrandom or clustered—a condition that has been demonstrated mathematically to increase the transmission potential of malaria. Finally, by matching the genetic profiles of the mosquito blood meals to that of the village residents, individuals and their sex and age group who were frequently fed on by the mosquitoes were identified. These information about biased feeding by vectors are useful when considering targeted malaria intervention.

## Supplemental Material


Supplemental materials
