# Supplementary material for: Nonrandom Selection and Multiple Blood Feeding of Human Hosts by Anopheles Vectors: Implications for Malaria Transmission in Papua New Guinea
Source: Am J Trop Med Hyg. 2021 Sep 27;105(6):1747–58. doi: 10.4269/ajtmh.21-0210 (PMC8641310; doi:10.4269/ajtmh.21-0210)
Supplement: Supplementary file 1 [file tpmd210210.SD1.pdf]

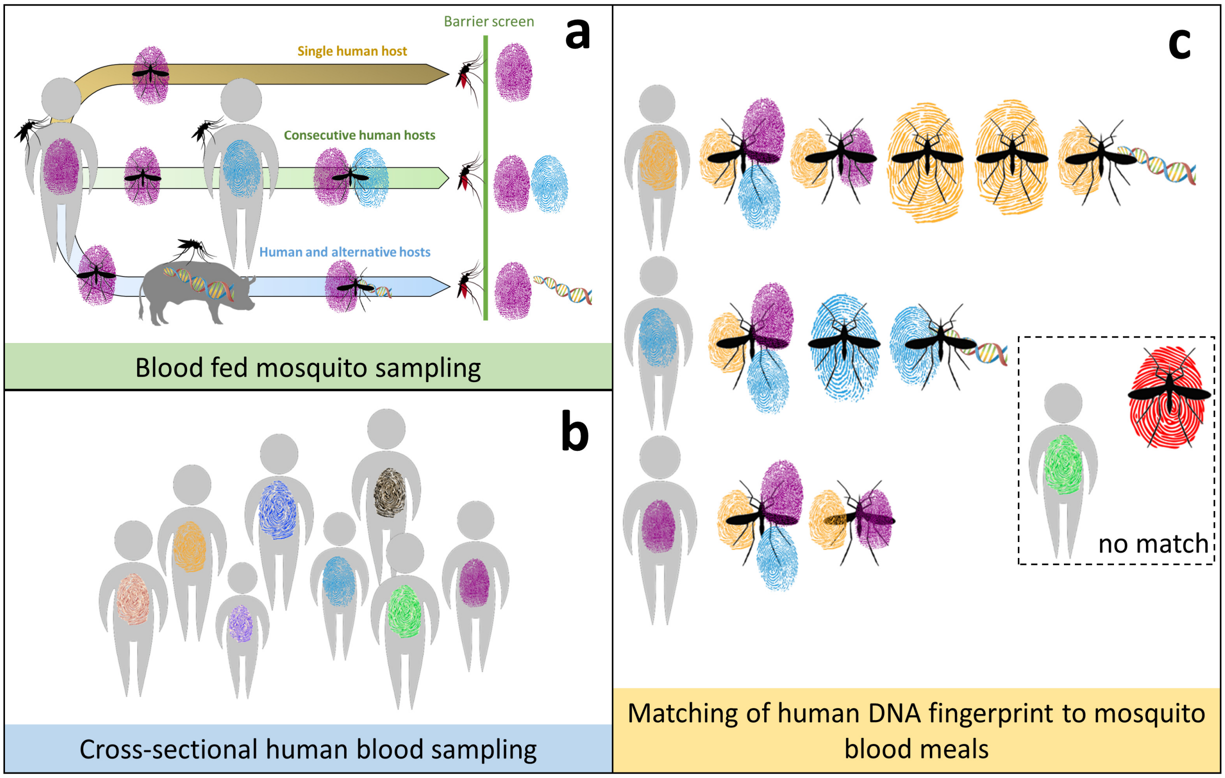

Figure S1. Schematic presentation of the study methodology. (a) Mosquitoes with different feeding histories (e.g., single feed on one human individual; feeds on multiple individuals; feeds on a human and an alternative hosts) are collected on barrier screens and their blood meals are genotyped to obtain the human genetic profile ("DNA fingerprint"). (b) Human individuals are blood-sampled at the same time as the mosquitoes are collected and their genetic profiles are analyzed. (c) Genetic profiles obtained from human individuals and mosquito blood meals are matched to determine patterns of host utilization.

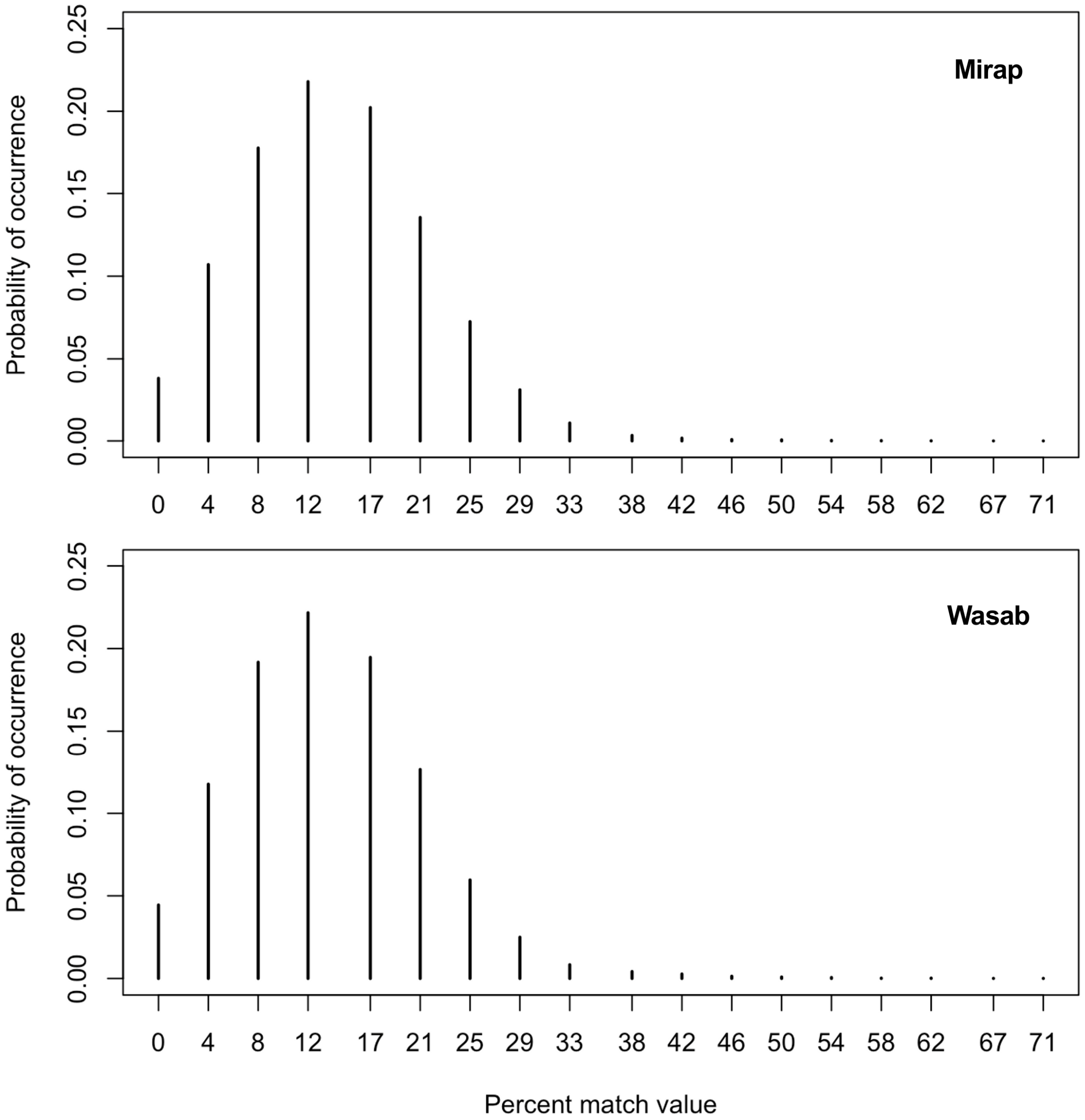

Figure S2. Probability of occurrence (y axis) of different percent match values (x axis) calculated from pairwise percent profile match analysis of 419 genetic profiles of Mirap (top panel) and 164 genetic profiles of Wasab (bottom panel) residents.

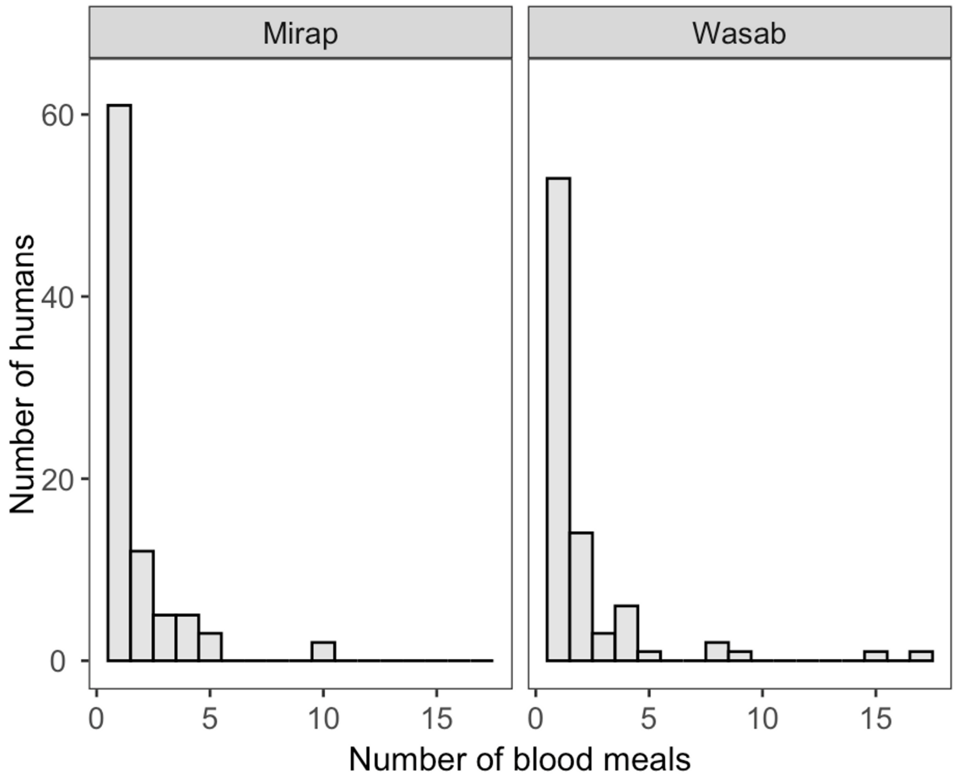

Figure S3. Histogram showing the number of blood meals taken on a human (x axis) versus the number of different human individuals with a particular blood meal frequency (y axis) for *Anopheles* mosquitoes in Mirap and Wasab village.

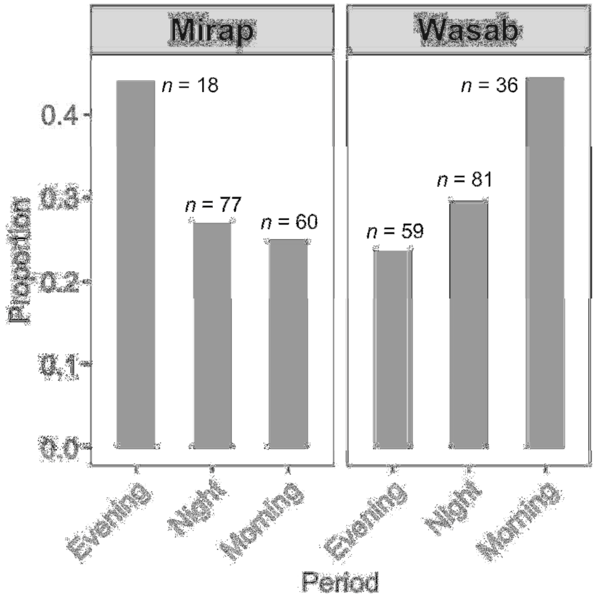

Figure S4. Bar plot showing proportion of female-fed blood meals relative to male-fed ones in blood-fed samples of *Anopheles* spp. collected in the evening (6pm–10pm), night (10pm–2am) and morning (2am–6am) period of the night in Mirap and Wasab villages. Numbers above the bars are sample sizes (*n*) of the blood-fed mosquitoes. The observed variation in the proportion of female-fed blood meals relative to male-fed ones among the three periods of the night was not significant in Mirap ($\chi^2 = 2.67$, df = 2, $P = 0.26$) or Wasab ($\chi^2 = 4.59$, df = 2, $P = 0.1$).
